# A novel variant in the *FLCN* gene in a Chinese family with Birt–Hogg–Dubé syndrome

**DOI:** 10.1002/mgg3.2488

**Published:** 2024-07-04

**Authors:** He Miao, Yulin Zhou, Silun Ge, Yufeng Gu, Le Qu, Wenquan Zhou, Haowei He

**Affiliations:** ^1^ Department of Urology, Jinling Hospital, Jinling School of Clinical Medicine Nanjing Medical University Nanjing Jiangsu China; ^2^ Department of Urology, Affiliated Jinling Hospital Medical School of Nanjing University Nanjing Jiangsu China

**Keywords:** Birt–Hogg–Dubé, *FLCN*, genetic, renal tumor, variant

## Abstract

**Background:**

This study aimed to identify disease‐causing variants within a Chinese family affected by Birt–Hogg–Dubé syndrome (BHDS), which arises from an autosomal dominant inheritance pattern attributed to variants in the folliculin (*FLCN*) gene, recognized as a tumor suppressor gene.

**Methods:**

A Chinese proband diagnosed with BHDS due to renal tumors underwent next‐generation sequencing (NGS), revealing a novel variant in the *FLCN* gene. Sanger sequencing was subsequently performed on blood samples obtained from family members to confirm the presence of this variant.

**Results:**

A novel germline frameshift variant (NM_144997.5:c.977dup) was identified in five individuals among the screened family members, marking the first report of this variant. Additionally, a somatic frameshift variant (NM_144997.5:c.1252del) was detected in the renal tumors of the proband. No variant was detected in unaffected family members.

**Conclusions:**

A novel heterozygous variant was identified in exon 9 of the *FLCN* gene, which broadens the spectrum of *FLCN* variants. We recommend that molecular analysis of the *FLCN* gene be performed in patients with suspected BHDS and their families.

## Graphical abstract

In a BHD‐associated renal tumors Chinese family. A novel heterozygous variant (NM_144997.5:c.977dup) was identified in exon 9 of the FLCN gene, which broadens the spectrum of FLCN variants. We recommend that molecular analysis of the FLCN gene be performed in patients with suspected BHDS and their families.

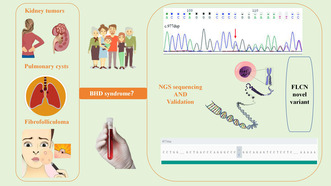

## 1 INTRODUCTION

Birt–Hogg–Dubé syndrome (BHDS) is a rare autosomal dominant disorder, initially proposed and named in 1977. It is caused by germline variants in the *FLCN* gene (Gene MIM number: 607273) and located on the short arm of chromosome 17p11.2 (Birt et al., 1977). The penetrance of BHDS is generally high, although the disease's expression varies significantly among family members and across different families (Schmidt et al., 2005). Typical clinical manifestations include multiple pulmonary cysts (recurrent spontaneous pneumothorax), cutaneous fibrofolliculoma, and various histological types of renal tumors. Literature indicates that approximately 16%–30% of BHDS patients will develop renal tumors, typically around the age of 50, which is seven times higher than the general population. Renal tumor development usually occurs later than skin and lung symptoms (Hasumi et al., 2016). The disease exhibits wide phenotypic heterogeneity, and clear genotype–phenotype correlations have not yet been established (Furuya et al., 2020; Sattler et al., 2018). Notably, skin manifestations are often less prominent in Asian individuals, and pulmonary cysts may go unnoticed (Iwabuchi et al., 2018). Furthermore, due to insufficient awareness of the disease, physicians often treat each symptom individually, potentially overlooking the presence of BHDS, leading to a high rate of misdiagnosis and missed diagnoses. Thus, while BHDS is widely considered highly variable in its phenotype, this interpretation may slightly deviate from the actual clinical scenario.

In this study, we examined clinical and genetic data from a patient with BHDS‐associated renal tumors in China and identified a novel variant in the *FLCN* gene region, which was confirmed by Sanger sequencing of her large family members. Our results provide genetic counseling for this family and enrich the *FLCN* gene mutation database, contributing to the exploration of the relationship between BHDS genotype and phenotype.

## 2 MATERIALS AND METHODS

### 2.1 Ethical compliance

The study was approved by the institutional review board of initiating center Jinling Hospital (ID Number: 2021NZKY‐004‐01). Written informed consent was obtained from all participants.

### 2.2 Patient recruitment and evaluation

A 35‐year‐old female proband was diagnosed with a high suspicion of BHDS at Jinling Hospital. A retrospective analysis of her clinical and pathological data was conducted, and the family history of 21 relatives across four generations was collected. Written informed consent was obtained from all individuals for potential publication of identifiable images or data in this study. All subjects underwent skin screening by professional dermatologists. Computed tomography (CT) of the chest was performed to evaluate the pulmonary lesions, and abdominal ultrasound examinations were conducted to rule out renal involvement.

### 2.3 Identification by next‐generation sequencing

With their full notification and informed consent, peripheral blood and tumor samples from the proband were drawn and sent to a third‐party company for sequencing. First, the DNA was fragmented, and the library prepared. Then, the entire coding regions including the intronic flanking sequences of *FLCN* were amplified by PCR. The software IDT2 was used to design primers for PCR. Sequences of PCR products were determined by the ABI 3100 Genetic Analyzer (Thermo Fisher Scientific, Inc., Waltham, MA, United States). Finally, The DNA sequencing reaction of the proband was performed using the next‐generation sequencing (NGS) method according to manufacturer's protocols. The multiple *FLCN* protein sequences were aligned using the program MUSCLE (version 3.6). The online databases, PolyPhen‐2 (polymorphism phenotyping), and MutationTaster programs were used to predict the possible effects of variants on the function of the proteins. Please refer to the American College of Medical Genetics and Genomics (ACMG) for standards and guidelines on the interpretation of genetic variations. The GenBank reference sequence and version number for the *FLCN* transcript variant was NM_144997.5. The *FLCN* protein sequence and confirmed by use of the Mutalyzer program (http://www.lovd.nl/mutalyzer).

### 2.4 Validation by Sanger sequencing

To validate the novel variants identified by NGS, 5 mL of peripheral blood was drawn from the proband's family members for Sanger sequencing to confirm the presence or absence of these variants. The sequencing products were purified and analyzed by Wuhan Yingjun Biotechnology Service Co., Ltd., and the results were interpreted using Chromas software.

## 3 RESULTS

### 3.1 Clinical characteristics

**FIGURE 1:**
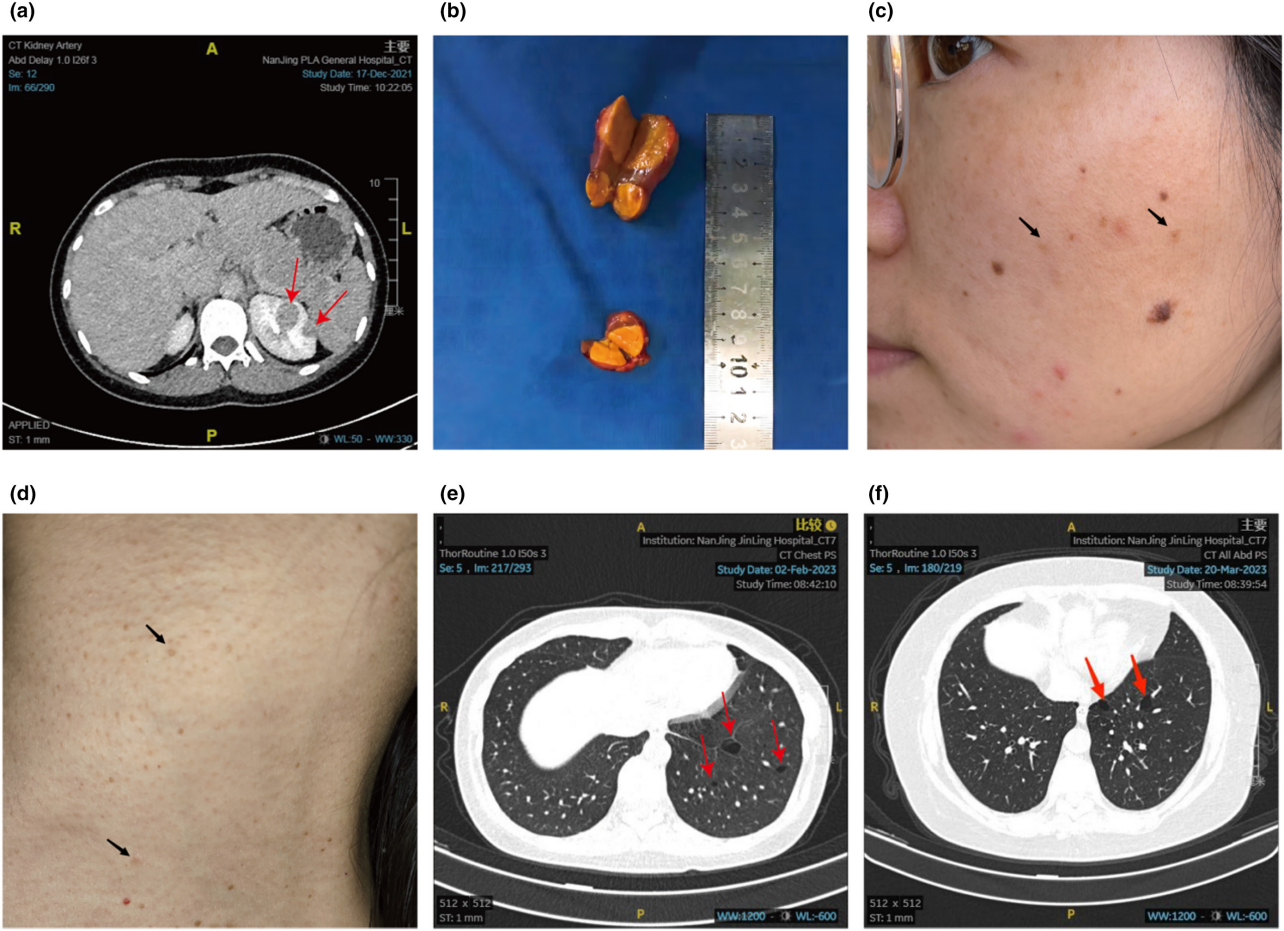
Clinical characteristics of the proband and her aunt. (a) Computed tomography of the proband's abdomen. The red arrow indicates multiple tumors in the left kidney. (b) Three renal tumors of the proband, ranging in diameter from 1.5 to 3 cm, after partial nephrectomy. (c) Multiple fibrous papules on the proband's face. (d) Multiple fibrous papules on the proband's neck. (e) Computed tomography of the proband's chest indicated multiple pulmonary bullae on the left side. (f) Chest computed tomography image of the proband's aunt showed multiple pulmonary bullae.

**FIGURE 2:**
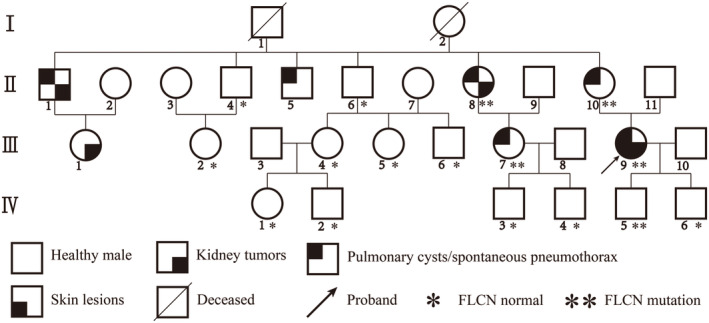
Family pedigree. Each closed quarter of rectangle and quadrant indicates a manifestation of BHDS in the figure. Four family members have developed kidney tumors (II1;II8;III1;III9). Only the proband (III9) exhibited cutaneous lesions. Sixteen individuals underwent *FLCN* gene sequencing, and five were detected to carry a germline frameshift variant.

In our study, the proband (III9) underwent robot‐assisted laparoscopic partial nephrectomy surgery due to multiple left renal tumors. Surgery resulted in the complete resection of three tumors, ranging in diameter from approximately 1.5 cm to 3 cm (Figure 1a,b). Physical examination revealed multiple papules and brown freckles on her face and neck (Figure 1c,d), which are highly suggestive of BHDS fibrofolliculoma‐like changes. However, she declined a further skin biopsy. Chest CT revealed bilateral lung cysts (Figure 1e), and she had been diagnosed with spontaneous pneumothorax at the age of 29. In her family, her grandfather (I1) died of gastric carcinoma, and her grandmother (I2) died of pancreatic adenocarcinoma without any symptoms of renal tumors. Seven family members, including the proband, developed BHDS‐related symptoms. Specifically, her first uncle (II1) had multiple pulmonary cysts and renal tumors, leading to a radical nephrectomy. His daughter (III1) underwent partial nephrectomy for renal tumors several years later. The proband's mother (II10) and her third uncle (II5) both experienced spontaneous pneumothorax, and subsequent chest CT confirmed multiple pulmonary lesions. One aunt (II8) underwent radical nephrectomy due to renal tumor and also had lung cysts (Figure 1f), while her daughter (III7) was diagnosed with multiple lung cysts following pneumothorax. None of the affected patients, except for the proband, exhibited cutaneous lesions. None of her father's relatives presented any symptoms of BHDS. Patients who underwent surgery were followed up through outpatient or telephone visits and showed no signs of tumor metastasis or recurrence. The clinical characteristics of the living family members are summarized in Table 1. The pedigree of the family members included in the study is shown in Figure 2.

**TABLE 1:** Clinical information and genotypes of individuals from the Chinese family with BHDS.

Patient No.	Age (years)	Gender	Affected or not	FLCN genotype	Manifestations and age of onset (years)			Treatment
					Lung lesions	Renal tumors	Skin lesions	
II1	64	M	Y	NA	Y (27)	Y (41)	N	RN
II4	67	M	N	Normal	N	N	N	N
II5	53	M	Y	NA	Y (31)	N	N	N
II6	58	M	N	Normal	N	N	N	N
II8	57	F	Y	c.977dup	Y (29)	Y (53)	N	RN
II10	62	F	Y	c.977dup	Y (46)	N	N	N
III1	36	F	Y	NA	N	Y (33)	N	PN
III2	45	F	N	Normal	N	N	N	N
III4	36	F	N	Normal	N	N	N	N
III5	22	F	N	Normal	N	N	N	N
III6	14	M	N	Normal	N	N	N	N
III7	34	F	Y	c.977dup	Y (30)	N	N	N
III9	35	F	Y	c.977dup	Y (29)	Y (35)	Y (20)	PN
IV1	12	F	N	Normal	N	N	N	N
IV2	10	M	N	Normal	N	N	N	N
IV3	11	M	N	Normal	N	N	N	N
IV4	4	M	N	Normal	N	N	N	N
IV5	13	M	Y	c.977dup	N	N	N	N
IV6	8	M	N	Normal	N	N	N	N

*Note*: Transcript number:NM_144997.5.

Abbreviations: F, female; M, male; N, no; NA, not available; PN, partial nephrectomy; RN, radical nephrectomy; Y, YES.

### 3.2 Histopathologic analysis

**FIGURE 3:**
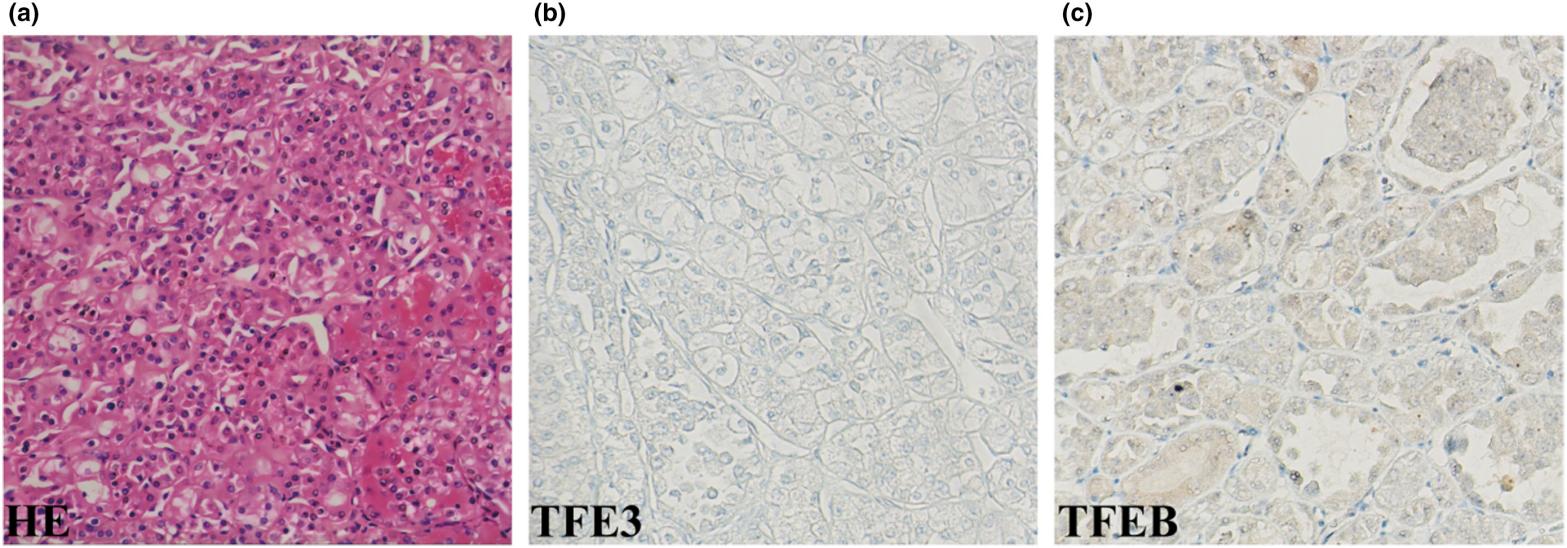
Pathological staining images of renal tumors in the proband (×200). (a) Hematoxylin–eosin staining of tumors. Immunohistochemical analyses for Transcription Factor Binding To IGHM Enhancer 3 (TFE3) (b), Transcription factor EB (TFEB) (c).

The proband's specimen exhibited a plant‐like arrangement of cells resembling chromophobe renal cell carcinoma, but lacked the characteristic loose stroma, central scar, and kidney‐shaped growth pattern (Figure 3). The pathological diagnosis of the multinodular tumors was hybrid oncocytic/chromophobe tumor (HOCT). Immunohistochemical analysis revealed positive staining for Pax‐8 (3+), Cathepsin‐K (2+), CK7 (scattered+), CD117 (2+), FH (3+), SDHB (2+), E‐cad (2+), and Ki‐67 (about 5%+). Negative staining was observed for P504s, CK20, TFE3, CA9, TFEB, Melan‐A, HMB‐45, CD10, and Vimentin. The expression of PD‐L1 was high, with a Tumor Proportion Score (TPS) of 65%. The proband's aunt and cousin were also diagnosed with HOCT, while her first uncle was diagnosed with chromophobe renal cell carcinoma (chRCC).

### 3.3 *FLCN* gene variation analysis

**FIGURE 4:**
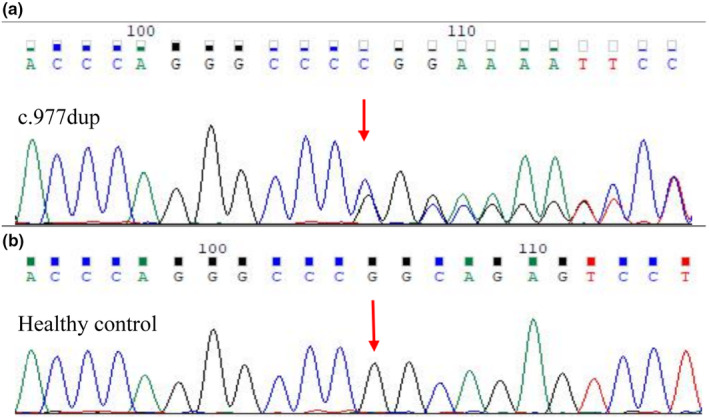
Sequence analysis of the *FLCN* gene. (a) The chromatograms show a novel heterozygous frameshift variation (red arrow) of c.977dup in the proband; (b) Sequencing result of a normal sequence in the unaffected son.

A germline frameshift variant was identified in exon 9 of the *FLCN* gene (NM_144997.5:c.977dup) in the proband. The variant causes an alteration of alanine to glycine at position 327 of the coding protein, resulting in premature termination at position 63 and truncation of the polypeptide chain. Five out of 16 relatives were found to carry the same *FLCN* gene germline variant (II8;II10;III7;III9;IV5), indicating that this familial disorder is caused by the *FLCN* gene germline variant (Figure 4a,b). The *FLCN* variant (p.Ala327GlyfsTer63) has never been previously reported in ClinVar, LOVD, or VarCards genetic variation databases, nor in the 1000_CN, ESP6500, or other population databases. It is also not included in the ClinVar database and *FLCN* mutation database (http://www.lovd.nl/FLCN). According to ACMG guidelines, the variant was classified as potentially pathogenic. Additionally, a somatic frameshift variant (NM_144997.5:c.1252del) was also detected in the tumor cell DNA. This somatic variant is not cataloged in COSMIC v90 but is listed as pathogenic in LOVD. Notably, the tumor mutation burden (TMB) was 0.47 muts/Mb, and the microsatellite instability (MSI) status was microsatellite stable (MSS). No DNA copy‐number abnormalities were found. The NGS statistics for the proband's *FLCN* gene are presented in Table 2.

**TABLE 2:** Whole‐exome sequencing results of the FLCN gene in the proband (NM_144997.5).

Sample	FLCN	Mutation type	Nucleotide change	Amino acid change	Freq (%)	Chr	Exon
Blood and tumor	Germline	Frameshift	c.977dup	p.Ala327GlyfsTer63	47.5	17	9/14
Tumor	Somatic	Frameshift	c.1252del	p.Leu418TrpfsTer50	33.3	17	11/14

Abbreviations: Chr: chromosome; Freq, frequency.

## 4 DISCUSSION

BHDS is a rare autosomal dominant genetic disease, primarily reported in White populations. The prevalence of its common features may vary based on researchers' specialty and interests, making the accurate prevalence remain unclear. Renal tumors are a relatively infrequent manifestation of BHDS and distinct from sporadic renal cell carcinoma (RCC). A comprehensive review of 204 families with at least one manifestation of BHDS indicated that individuals with the *FLCN* variant have an estimated 19% risk of developing renal tumors in males and 21% in females (Bruinsma et al., 2023). While BHDS has been reported in several Asian families, particularly in Japan and Korea, most patients exhibit primarily pulmonary manifestations (Furuya et al., 2016; Hu et al., 2021; Liu et al., 2022). Only about 3.6% of BHDS patients in Asia present with renal tumors, possibly due to insufficient knowledge about BHDS among Asian physicians and potential racial differences. In our study, among the eight variant positive or clinically positive family members, four individuals (50%) developed renal tumors. This incidence rate is higher than the reported range of 16%–30% (Hasumi et al., 2016).

The *FLCN* gene encodes the highly conserved tumor suppressor folliculin, which interacts with folliculin‐interacting protein 1 and 2 (FNIP1 and FNIP2), as well as AMP‐activated protein kinase (AMPK), to regulate the mammalian target of rapamycin (mTOR) pathway. This pathway controls cell growth, proliferation, and survival. In a mice model (FLCN FLOX/FLOX/Ksp‐Cre), specific knockout of *FLCN* in renal distal tubule and collecting duct cells leads to polycystic kidney disease and uremia, resulting in death within 3 weeks of birth (Wu et al., 2015). Alterations in *FLCN* within renal distal tubular cells promote tumorigenesis and the development of various histological tumor phenotypes. The mTOR pathway plays a crucial role in the growth of isogeneic transplant models (Wu et al., 2015) with *FLCN* alterations and human UOK257 xenograft models. Variants in the *FLCN* gene diminish the tumor suppressor effect of these signaling pathways, triggering renal tumors in renal distal tubules in accordance with the second‐hit hypothesis (Vocke et al., 2005). Previous studies have reported the co‐occurrence of somatic variants in the *FLCN* gene in both BHDS‐associated renal tumors and parathyroid adenomas (Jha et al., 2023; Wang et al., 2022), which aligns with our findings and strongly supports this hypothesis (Daccord et al., 2020).

BHDS typically affects the skin, lungs, and kidneys. In rare cases, patients have also been reported to have colorectal cancer, salivary gland oncocytomas, melanomas, oral papules, and thyroid cancers, although it remains unclear whether *FLCN* variants are responsible for these manifestations (Daccord et al., 2020). Ethnicity appears to influence the presentation of BHDS. In North American and European patients, skin lesions are typically the predominant manifestation, followed by renal tumors at a later stage, with a low incidence of pneumothorax (Kluger et al., 2010). Conversely, BHDS patients in Asia often exhibit a “pulmonary phenotype,” characterized by multiple pulmonary cysts and recurrent spontaneous pneumothorax, while cutaneous manifestations are rare and less typical (Furuya et al., 2016, 2020).

The proband in our study exhibited a “renal‐phenotype” of BHDS, characterized by multiple renal tumors, while pulmonary and cutaneous manifestations were relatively nonspecific. The International BHD Consortium recommends diagnostic *FLCN* gene testing for BHDS patients starting at age 20 (Berger et al., 2020). Despite numerous studies on genotype–phenotype correlations in BHDS patients, no significant associations have been found thus far(Sattler et al., 2018), suggesting that a larger cohort may be necessary to uncover meaningful correlations.

According to the Leiden Open Variation Database, over 280 unique variants have been identified throughout the coding region of the *FLCN* gene to date (Lim et al., 2010). A recognized variant hotspot involves the deletion or insertion of cytosine residues in exon 11, with nearly half of patients carrying either c.1285delC or c.1285dupC variants, consistent across European and Asian populations (Schmidt et al., 2005). Other patients exhibit intragenic deletions or duplications and pathogenic missense variants (Benhammou et al., 2011; Matsumoto et al., 2021). A Japanese study (Furuya et al., 2016) revealed two additional variant hotspots, c.1533_1536delGATG and c.1347_1353dupCCACCCT, in the Japanese BHDS population. In our study, we identified a novel frameshift variant (c.977dup) in exon 9 of the *FLCN* gene, which has not been previously reported in the literature or the *FLCN* mutation database (Lim et al., 2010; Zhou et al., 2022).

BHDS‐associated renal tumors are typically bilateral and multifocal, characterized by low malignant potential but a lifetime risk of developing additional tumors (Daccord et al., 2020). It is important to differentiate it from other hereditary renal tumor syndromes, particularly tuberous sclerosis complex (TSC). BHDS‐associated renal tumors exhibit diverse histological features, with HOCT being the most common (approximately 50%), followed by chRCC (30%) and clear cell renal cell carcinoma (9%). Eosinophilic chromophobe carcinoma and papillary renal cell carcinoma are rare occurrences (Pavlovich et al., 2002). Preoperative diagnosis through imaging is challenging. For patients with HOCT, it is essential to conduct a detailed family history inquiry, perform chest CT to assess for pulmonary lesions, and carry out a thorough skin examination to determine the presence of BHDS. The 2015 diagnostic criteria for BHDS (Schmidt & Linehan, 2015) recommend the identification of *FLCN* gene germline variants through genetic testing as the optimal diagnostic standard. High‐precision DNA sequencing analysis is particularly important, with a detection rate exceeding 90%.

Currently, there are no specific treatments targeting the etiology of BHDS. Renal ultrasonography may not detect small or isoechoic HOCTs. Menko et al. (2009) recommend annual MRI scans for patients with BHDS starting at age 20 to screen for kidney cancer, although ultrasound can be used for preliminary screening, especially in institutions without access to MRI (Perdeaux & Solly, 2013). Most BHD‐associated renal tumors rarely metastasize at the time of initial diagnosis, and partial nephrectomy remains the primary treatment to preserve renal function. Jikuya et al. (2022) found that the HIF‐VEGF signaling pathway is not upregulated in the vascular system associated with BHD, indicating that angiogenesis inhibitors may not be effective treatments. The tumor of the proband showed strong expression of PD‐L1, suggesting that immune checkpoint inhibitors may have potential benefits, consistent with previous research findings. Additionally, Jikuya et al. (2022) discovered high expression of the mesenchymal to epithelial transition factor (MET) in various histological types of BHDS‐associated renal tumors, suggesting that MET inhibitors such as cabozantinib and crizotinib may provide promising therapeutic options for treating BHDS‐associated renal tumors.

In conclusion, we identified a novel heterozygous frameshift variant (NM_144997.5:c.977dup) in exon 9 of the *FLCN* gene. This discovery broadens the spectrum of *FLCN* gene variants in the Chinese population. Related functional studies examining the consequences of the identified *FLCN* gene variants should be conducted next, which could provide deeper insights into the pathogenesis of BHDS.

## AUTHOR CONTRIBUTIONS

He Miao collected the data and drafted the manuscript, while Le Qu and Yufeng Gu provided critical feedback and revisions. The data were analyzed by Silun Ge and Yulin Zhou. This study was designed by Haowei He and Wenquan Zhou. All authors read and approved the final version of the manuscript.

## FUNDING INFORMATION

National Natural Science Foundation of China, Grant/Award Number: 81972402 to Haowei He and 82072836 to Wenquan Zhou.

## CONFLICT OF INTEREST STATEMENT

The authors declare no conflicts of interest.

## ETHICS STATEMENT

The study was approved by the institutional review board of initiating center Jinling Hospital (ID Number: 2021NZKY‐004‐01). Informed written consent comprised DNA extraction, gene analysis, and CT examinations. Written informed consent was obtained from all participants, while the consent of the participants under 18 years old was signed by their parents.

## CONSENT FOR PUBLICATION

Written informed consent was obtained from all patients to publish their cases in this study.

## Data Availability

The next‐generation sequencing raw data have been deposited in Sequence Read Archive database (accession number: SRR26320486). The data that support the findings of this study are available from the corresponding author upon reasonable request.

## References

[mgg32488-bib-0001] Benhammou, J. N. , Vocke, C. D. , Santani, A. , Schmidt, L. S. , Baba, M. , Seyama, K. , Wu, X. , Korolevich, S. , Nathanson, K. L. , Stolle, C. A. , & Linehan, W. M. (2011). Identification of intragenic deletions and duplication in the FLCN gene in Birt‐Hogg‐Dubé syndrome. Genes, Chromosomes & Cancer, 50(6), 466–477. 21412933 10.1002/gcc.20872 PMC3075348

[mgg32488-bib-0002] Berger, I. , Berland, S. , Rodriguez, J. R. , Aamodt, H. , Sitek, J. C. , Jørgensen, K. , & Johansen, T. E. B. (2020). Birt‐Hogg‐Dubé syndrome. Tidsskrift for den Norske laegeforening: tidsskrift for praktisk medicin, ny raekke, 140(6), 1–8. 10.4045/tidsskr.18.0848 32321218

[mgg32488-bib-0003] Birt, A. R. , Hogg, G. R. , & Dubé, W. J. (1977). Hereditary multiple fibrofolliculomas with trichodiscomas and acrochordons. Archives of Dermatology, 113(12), 1674–1677. 596896

[mgg32488-bib-0004] Bruinsma, F. J. , Dowty, J. G. , Win, A. K. , Goddard, L. C. , Agrawal, P. , Attina', D. , Bissada, N. , de Luise, M. , Eisen, D. B. , Furuya, M. , Gasparre, G. , Genuardi, M. , Gerdes, A. M. , Hansen, T. V. O. , Houweling, A. C. , Johannesma, P. C. , Lencastre, A. , Lim, D. , Lindor, N. M. , … Genetic Susceptibility Working Group I‐CONFIRM . (2023). Update of penetrance estimates in Birt‐Hogg‐Dubé syndrome. Journal of Medical Genetics, 60(4), 317–326. 36849229 10.1136/jmg-2022-109104

[mgg32488-bib-0005] Daccord, C. , Good, J. M. , Morren, M. A. , Bonny, O. , Hohl, D. , & Lazor, R. (2020). Birt‐Hogg‐Dubé syndrome. European Respiratory Review: An Official Journal of the European Respiratory Society, 29(157), 200042. 32943413 10.1183/16000617.0042-2020 PMC9489184

[mgg32488-bib-0006] Furuya, M. , Hasumi, H. , Yao, M. , & Nagashima, Y. (2020). Birt‐Hogg‐Dubé syndrome‐associated renal cell carcinoma: Histopathological features and diagnostic conundrum. Cancer Science, 111, 15–22. 31777168 10.1111/cas.14255 PMC6942440

[mgg32488-bib-0007] Furuya, M. , Yao, M. , Tanaka, R. , Nagashima, Y. , Kuroda, N. , Hasumi, H. , Baba, M. , Matsushima, J. , Nomura, F. , & Nakatani, Y. (2016). Genetic, epidemiologic and clinicopathologic studies of Japanese Asian patients with Birt‐Hogg‐Dubé syndrome. Clinical Genetics, 90(5), 403–412. 27220747 10.1111/cge.12807

[mgg32488-bib-0008] Hasumi, H. , Baba, M. , Hasumi, Y. , Furuya, M. , & Yao, M. (2016). Birt‐Hogg‐Dubé syndrome: Clinical and molecular aspects of recently identified kidney cancer syndrome. International Journal of Urology: Official Journal of the Japanese Urological Association, 23(3), 204–210. 26608100 10.1111/iju.13015

[mgg32488-bib-0009] Hu, X. , Zhang, G. , Chen, X. , & Xu, K. F. (2021). Birt‐Hogg‐Dubé syndrome in Chinese patients: A literature review of 120 families. Orphanet Journal of Rare Diseases, 16(1), 223. 34001170 10.1186/s13023-021-01848-8 PMC8130425

[mgg32488-bib-0010] Iwabuchi, C. , Ebana, H. , Ishiko, A. , Negishi, A. , Mizobuchi, T. , Kumasaka, T. , Kurihara, M. , & Seyama, K. (2018). Skin lesions of Birt‐Hogg‐Dubé syndrome: Clinical and histopathological findings in 31 Japanese patients who presented with pneumothorax and/or multiple lung cysts. Journal of Dermatological Science, 89(1), 77–84. 29157599 10.1016/j.jdermsci.2017.10.014

[mgg32488-bib-0011] Jha, S. , Welch, J. , Tora, R. , Lack, J. , Warner, A. , del Rivero, J. , Sadowski, S. M. , Nilubol, N. , Schmidt, L. S. , Linehan, W. M. , Weinstein, L. S. , Simonds, W. F. , & Agarwal, S. K. (2023). Germline‐ and somatic‐inactivating FLCN variants in parathyroid cancer and atypical parathyroid tumors. The Journal of Clinical Endocrinology and Metabolism, 108(10), 2686–2698. 36935552 10.1210/clinem/dgad136 PMC10505536

[mgg32488-bib-0012] Jikuya, R. , Murakami, K. , Nishiyama, A. , Kato, I. , Furuya, M. , Nakabayashi, J. , Ramilowski, J. A. , Hamanoue, H. , Maejima, K. , Fujita, M. , Mitome, T. , Ohtake, S. , Noguchi, G. , Kawaura, S. , Odaka, H. , Kawahara, T. , Komeya, M. , Shinoki, R. , Ueno, D. , … Hasumi, H. (2022). Single‐cell transcriptomes underscore genetically distinct tumor characteristics and microenvironment for hereditary kidney cancers. iScience, 25(6), 104463. 35874919 10.1016/j.isci.2022.104463 PMC9301876

[mgg32488-bib-0013] Kluger, N. , Giraud, S. , Coupier, I. , Avril, M. F. , Dereure, O. , Guillot, B. , Richard, S. , & Bessis, D. (2010). Birt‐Hogg‐Dubé syndrome: Clinical and genetic studies of 10 French families. The British Journal of Dermatology, 162(3), 527–537. 19785621 10.1111/j.1365-2133.2009.09517.x

[mgg32488-bib-0014] Lim, D. H. , Rehal, P. K. , Nahorski, M. S. , Macdonald, F. , Claessens, T. , Van Geel, M. , Gijezen, L. , Gille, J. J. , Giraud, S. , Richard, S. , van Steensel, M. , Menko, F. H. , & Maher, E. R. (2010). A new locus‐specific database (LSDB) for mutations in the folliculin (FLCN) gene. Human Mutation, 31(1), E1043–E1051. 19802896 10.1002/humu.21130

[mgg32488-bib-0015] Liu, S. , Xia, K. , Liu, X. , Duan, Y. , Hu, M. , Xia, H. , Lv, J. , Zhang, L. , Liu, Y. , Xia, X. , Li, G. , & Cui, X. (2022). Birt‐Hogg‐Dubé syndrome. Frontiers in Medicine, 9:857127. 10.3389/fmed.2022.857127 PMC9035795 35479937

[mgg32488-bib-0016] Matsumoto, K. , Lim, D. , Pharoah, P. D. , Maher, E. R. , & Marciniak, S. J. (2021). A systematic review assessing the existence of pneumothorax‐only variants of FLCN. Implications for lifelong surveillance of renal tumours. European Journal of Human Genetics: EJHG, 29(11), 1595–1600. 34267338 10.1038/s41431-021-00921-x PMC8560836

[mgg32488-bib-0017] Menko, F. H. , Van Steensel, M. A. , Giraud, S. , Friis‐Hansen, L. , Richard, S. , Ungari, S. , Nordenskjöld, M. , Hansen, T. V. , Solly, J. , Maher, E. R. , & European BHD Consortium . (2009). Birt‐Hogg‐Dubé syndrome: diagnosis and management. The Lancet Oncology, 10(12), 1199–1206. 19959076 10.1016/S1470-2045(09)70188-3

[mgg32488-bib-0018] Pavlovich, C. P. , Walther, M. M. , Eyler, R. A. , Hewitt, S. M. , Zbar, B. , Linehan, W. M. , & Merino, M. J. (2002). Renal tumors in the Birt‐Hogg‐Dubé syndrome. The American Journal of Surgical Pathology, 26(12), 1542–1552. 12459621 10.1097/00000478-200212000-00002

[mgg32488-bib-0019] Perdeaux, E. , & Solly, J. (2013). Birt‐Hogg‐Dubé syndrome. JAMA, 309(14), 1460. 10.1001/jama.2013.2243 23571576

[mgg32488-bib-0020] Sattler, E. C. , Reithmair, M. , & Steinlein, O. K. (2018). Kidney cancer characteristics and genotype–phenotype‐correlations in Birt‐Hogg‐Dubé syndrome. PLoS One, 13(12), e0209504. 30586397 10.1371/journal.pone.0209504 PMC6306193

[mgg32488-bib-0021] Schmidt, L. S. , & Linehan, W. M. (2015). Molecular genetics and clinical features of Birt‐Hogg‐Dubé syndrome. Nature Reviews. Urology, 12(10), 558–569. 26334087 10.1038/nrurol.2015.206 PMC5119524

[mgg32488-bib-0022] Schmidt, L. S. , Nickerson, M. L. , Warren, M. B. , Glenn, G. M. , Toro, J. R. , Merino, M. J. , Turner, M. L. , Choyke, P. L. , Sharma, N. , Peterson, J. , Morrison, P. , Maher, E. R. , Walther, M. C. M. , Zbar, B. , & Linehan, W. M. (2005). Germline BHD‐mutation spectrum and phenotype analysis of a large cohort of families with Birt‐Hogg‐Dubé syndrome. American Journal of Human Genetics, 76(6), 1023–1033. 15852235 10.1086/430842 PMC1196440

[mgg32488-bib-0023] Vocke, C. D. , Yang, Y. , Pavlovich, C. P. , Schmidt, L. S. , Nickerson, M. L. , Torres‐Cabala, C. A. , Merino, M. J. , Walther, M. C. M. , Zbar, B. , & Linehan, W. M. (2005). High frequency of somatic frameshift BHD gene mutations in Birt‐Hogg‐Dubé‐associated renal tumors. Journal of the National Cancer Institute, 97(12), 931–935. 15956655 10.1093/jnci/dji154

[mgg32488-bib-0024] Wang, T. , Yang, Y. , Feng, H. , Cui, B. , Lv, Z. , Zhao, W. , Zhang, X. , & Ma, X. (2022). Concurrent germline and somatic mutations in FLCN and preliminary exploration of its function: A case report. Frontiers in Oncology, 12, 877470. 35664771 10.3389/fonc.2022.877470 PMC9162506

[mgg32488-bib-0025] Wu, M. , Si, S. , Li, Y. , Schoen, S. , Xiao, G. Q. , Li, X. , Teh, B. T. , Wu, G. , & Chen, J. (2015). Flcn‐deficient renal cells are tumorigenic and sensitive to mTOR suppression. Oncotarget, 6(32), 32761–32773. 26418749 10.18632/oncotarget.5018 PMC4741728

[mgg32488-bib-0026] Zhou, W. , Liu, K. , Xu, K. F. , Liu, Y. , & Tian, X. (2022). Clinical and genetic comparison of Birt‐Hogg‐Dubé syndrome (Hornstein‐Knickenberg syndrome) in Chinese: A systemic review of reported cases. International Journal of General Medicine, 15, 5111–5121. 35637701 10.2147/IJGM.S359660 PMC9144823

